# Integration of protein context improves protein-based COVID-19 patient stratification

**DOI:** 10.1186/s12014-022-09370-0

**Published:** 2022-08-11

**Authors:** Jinlong Gao, Jiale He, Fangfei Zhang, Qi Xiao, Xue Cai, Xiao Yi, Siqi Zheng, Ying Zhang, Donglian Wang, Guangjun Zhu, Jing Wang, Bo Shen, Markus Ralser, Tiannan Guo, Yi Zhu

**Affiliations:** 1 Westlake Laboratory of Life Sciences and Biomedicine, Key Laboratory of Structural Biology of Zhejiang Province, School of Life Sciences, Westlake University, Hangzhou, Zhejiang China; 2 Institute of Basic Medical Sciences, Westlake Institute for Advanced Study, Hangzhou, Zhejiang China; 3 Taizhou Hospital, Wenzhou Medical University, Linhai, Zhejiang China; 4 Molecular Biology of Metabolism Laboratory, The Francis Crick Institute, London, UK; 5 Department of Biochemistry, Charité - Universitätsmedizin Berlin, corporate member of Freie Universität Berlin and Humboldt-Universität Zu Berlin, Berlin, Germany

**Keywords:** COVID-19, Severe cases, Proteomics, Protein complex, Stoichiometric ratio

## Abstract

**Background:**

Classification of disease severity is crucial for the management of COVID-19. Several studies have shown that individual proteins can be used to classify the severity of COVID-19. Here, we aimed to investigate whether integrating four types of protein context data, namely, protein complexes, stoichiometric ratios, pathways and network degrees will improve the severity classification of COVID-19.

**Methods:**

We performed machine learning based on three previously published datasets. The first was a SWATH (sequential window acquisition of all theoretical fragment ion spectra) MS (mass spectrometry) based proteomic dataset. The second was a TMTpro 16plex labeled shotgun proteomics dataset. The third was a SWATH dataset of an independent patient cohort.

**Results:**

Besides twelve proteins, machine learning also prioritized two complexes, one stoichiometric ratio, five pathways, and five network degrees, resulting a 25-feature panel. As a result, a model based on the 25 features led to effective classification of severe cases with an AUC of 0.965, outperforming the models with proteins only. Complement component C9, transthyretin (TTR) and TTR-RBP (transthyretin-retinol binding protein) complex, the stoichiometric ratio of SAA2 (serum amyloid A proteins 2)/YLPM1 (YLP Motif Containing 1), and the network degree of SIRT7 (Sirtuin 7) and A2M (alpha-2-macroglobulin) were highlighted as potential markers by this classifier. This classifier was further validated with a TMT-based proteomic data set from the same cohort (test dataset 1) and an independent SWATH-based proteomic data set from Germany (test dataset 2), reaching an AUC of 0.900 and 0.908, respectively. Machine learning models integrating protein context information achieved higher AUCs than models with only one feature type.

**Conclusion:**

Our results show that the integration of protein context including protein complexes, stoichiometric ratios, pathways, network degrees, and proteins improves phenotype prediction.

**Supplementary Information:**

The online version contains supplementary material available at 10.1186/s12014-022-09370-0.

## Background

COVID-19 caused by SARS-CoV-2 remains an ongoing pandemic [1]. Distinguishing severe and non-severe cases is crucial since only the severe cases require special treatment such as artificial ventilation [2]. Proteins from COVID-19 patients’ serum or plasma have been utilized to develop severity classifiers. In a TMT-based serum proteomics and metabolomics study, 118 sera samples from 65 COVID-19 patients and 53 controls were analyzed, resulting in a severity classifier based on characteristic proteins and metabolites [3]. Based on blood samples from early hospitalized cases, Messner et al. built a classifier including 27 proteins for the prediction of COVID-19 severity [4]. A longitudinal cohort from hospitalized COVID-19 patients identified a distinct proteomic trajectory associated with mortality in blood samples [5]. Another longitudinal cohort established an immune biomarker panel to gauge the severity of COVID-19 [6]. Demichev et al. presented a prognostic map of COVID-19 by linking clinical parameters to plasma proteomes [7] and established a proteomic survival predictor to distinguish severe cases [8]. A plasma-based proteomics study reported multiple modulated blood proteins of recovered COVID-19 patients 3 months after discharge [9]. Using the serum proteomics of COVID-19 patients with samples from different disease stages, Zhang et al. monitored disease progression and predicted viral nucleic acid positivity during COVID-19 [10].

However, all the above-mentioned protein classifiers for COVID-19 are based on individual biomolecules, mainly proteins, ignoring the fact that no protein functions in an isolated manner. As the ultimate effectors of diseases, protein complexes regulate many core biological processes. For example, mitochondrial complexes, such as the mitochondrial ribosomal small subunit, respiratory chain complex I and the mitochondrial pyruvate dehydrogenase complex, are involved in energy production and found to be highly conserved but dysregulated in diseases such as cancers [11]. Several tools for analyzing the protein complex features have been reported in recent years, including NetProt and Fuzzy-FishNET [12, 13]. PCprophet is a machine learning based software for identifying protein complex [14]. Protein stoichiometric ratios in a complex are also important since they are self-normalized and relatively conserved [15]. In addition, protein network degree is the number of edges connected to a protein in a network that includes all proteins in the matrix. Network degree reflects expression associations with other proteins [16]. In an in vitro cell culture experiment, 332 high-confidence protein–protein interactions between SARS-CoV-2 and host proteins were identified using affinity purification mass spectrometry, screening out two sets of pharmacological agents [17]. However, this study is limited to in vitro cell line culture, hence not directly transferrable to human plasma collected from patients with COVID-19.

As discussed above, although some studies have stratified COVID-19 severity based on protein levels, no protein functions in an isolated manner, and the information obtained from protein quantification alone may not be comprehensive. Besides the abundance, multidimensional information of proteins, including but not limited to protein complexes, protein topology, post translational modifications, etc., are essential to understand the disease biology. In this study, we tried to investigate the aspect of protein complex using several COVID-19 datasets. We performed machine learning based on three previously published datasets [3, 4, 10]. The first was the training dataset, which was the MS data matrix generated using 20 min SWATH [10], containing 331 proteins, and these samples were obtained prior to the onset of disease severity. The second was the test dataset 1, which was the MS data matrix using 35 min TMTpro 16plex DDA proteomics [3], containing 894 proteins. The third was the test dataset 2, a MS data matrix analyzed by 5 min fast flow SWATH [4], containing 229 proteins. Via this way, we evaluated the feasibility to utilize protein context information, including protein complexes, stoichiometric ratios in a complex, protein pathways and degree of protein networks, besides proteins, as key features to classify severe COVID-19 cases. The results showed that protein context could be exploited as integrative biomarkers for the stratification of COVID-19.

## Methods

### Patients and samples

The training set for this study consisted of 54 sera samples from 40 Chinese patients with COVID-19 (25 non-severe and 15 severe, according to the Chinese Government Diagnosis and Treatment Guideline 5th version), which were quantified using SWATH MS based proteomics [10]. Its performance was subsequently evaluated in two test datasets. One was a TMTpro 16plex dataset from our previous publication [3], containing 21 samples from 21 Chinese patients (6 non-severe and 15 severe). The other was the SWATH data set of 102 sera samples from 31 German patients with COVID-19 [4].

### Serum sample collection, peptide preparation, and MS data acquisition

Regarding to the training set, the procedures for serum sample collection, peptides preparation and SWATH acquisition for the training set have been described in our previous study [10]. Briefly, these samples were collected from 40 patients with COVID-19 in stage 1, namely, the nucleic acid positive stage in the first 48 h after admission [10]. Most patients had only one blood test, while some of them had two blood tests as recorded in the medical history. In total, there were 54 sera samples collected from 40 patients.

Regarding to the test sets, the first test set used 35 min TMTpro 16plex proteomics. For each patient, the serum sample was obtained within 48 h after hospital admission [3]. The second test set used 5 min SWATH MS proteomics, and the samples were obtained from early hospitalized patients (nearly 1–2 days after hospital admission) [4]. The details of serum sample collection, peptide preparation and MS data acquisition in the two test sets have been described in the previously published studies [3, 4], respectively.

### Three proteomic data sets

The training set consisted of 54 sera samples from 40 Chinese COVID-19 patients, and resulted in a MS data matrix generated using 20 min SWATH, containing 331 proteins and 3474 peptides [10]. The test set 1 included 21 sera samples from 21 Chinese COVID-19 patients [3], and 894 proteins and 7747 peptides were identified from the MS data matrix using 35 min TMTpro 16plex DDA proteomics. The test set 2 contained 102 sera samples from 31 early hospitalized German patients, analyzed by 5 min fast flow SWATH [4], and the resultant data matrix included 229 proteins and 3000 peptides.

### The generation of protein complex, pathways, and stoichiometric ratios

The proteins in the training set of this study were from the dataset of previously published literature [10]. Subsequent features, including complexes, pathways, stoichiometric ratios and network degree, were all generated based on these proteins. Complexes were obtained from CORUM [18] and BioPlex Explorer 3.0 [19] using all proteins in the training cohort. Pathways were acquired by G:profiler (version e99_eg46_p14_f929183, database updated on 07/02/2020) from all proteins in the training cohort. The expression values of complexes and pathways were the sum of the Z-scores of proteins in complexes and pathways. The determination of Z-score was performed using the scale function of R package. In addition, stoichiometric ratios are the ratio of any two proteins in a complex. The values of stoichiometric ratios in a complex are the ratio of the two proteins treated with Z-score.

### The generation of network degree

The network degrees, which shows the degree to which one protein is related to another, were also generated based on proteins from dataset published before [10]. The value of a protein’s network degree is the sum of all its edge values. The edges mean the protein–protein expression associations, and the calculation of protein–protein association is based on a reference that calculates gene–gene association [20]. Briefly, the protein–protein association was determined by statistical independence of two proteins. The threshold for significant level was set as 0.01. If the normalized statistic of an equation was greater than the significant level, null hypothesis that proteins x and y are independent to each other were rejected, and the edge for x and y was equal to 1, otherwise it was equal to 0.

### The screening of differential features

The differential expression of proteins and other four features between severe cases and non-severe cases was determined using R package Limma (version 3.44) by fitting a linear model. Features with a p value < 0.05 were considered as differential features. It should be noted that although the adjustment of p values based on multiple hypothesis correction can avoid type-1 errors, the risk of introducing type-2 errors is also increased, eliminating some potentially differential proteins. Especially, when the sample size and number of proteins are not too large, there were few differential proteins left after p value adjustment, which was not enough for subsequent random forest machine learning model. Therefore, based on actual situation, multiple hypothesis correction was not performed in this study.

### The input variables and process of random forest machine learning

We used five categories of variables as the input for the machine learning model, including proteins, complexes, pathways, stoichiometric ratios in a complex, and network degrees. The values of all differential features were normalized by Z-score for machine learning. The output predictor of the machine learning model was the non-severe or severe disease type, and we built a random forest machine learning model for the binary classification task. The random forest model was based on an R package random forest (version 4.6-14) with 5000 trees and 5 nodes as the minimum size of terminal nodes, while the type of prediction was chosen to be probabilistic. The best features were selected by 100 times random forest machine learning. Ten-fold cross validation was performed for each training process of the machine learning model. Receiver Operator Curve (ROC) was estimated by predicting results of the cross-validation using R package pROC (version 1.15.3).

### Data visualization

PCAs were plotted using R package PCA. Heatmaps were plotted by R package heatmap (version 1.0.12). Density plots were performed by Kernel Density Estimation, a base function of R (version 4.0.0) (a Gaussian kernel with default bandwidth was used).

## Results

### Three sera proteomics data sets for modeling and testing

**Fig. 1:**
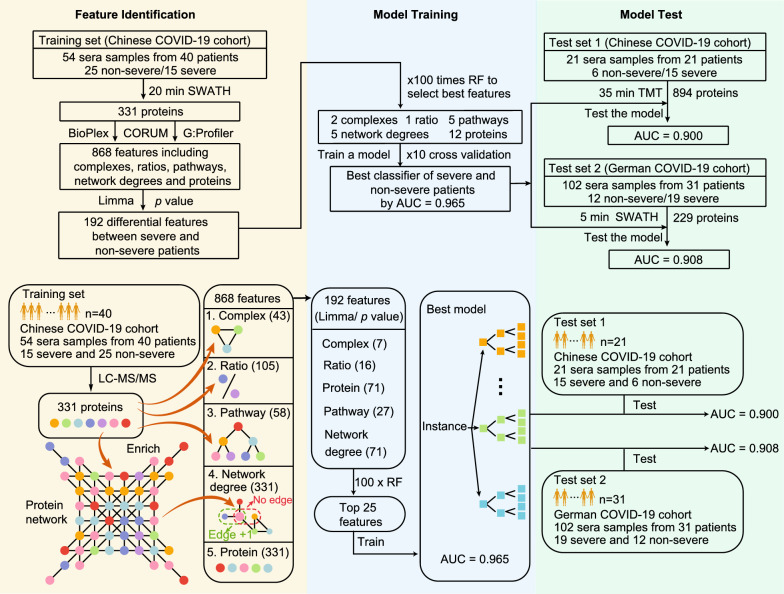
Study overview. In general, 331 proteins were identified from 54 serum samples of COVID-19 patients. Subsequently, five kinds of 868 features were derived from these proteins. The top 25 differential features were selected for the machine learning model, which was further validated in two test datasets

Three independently obtained proteomic data sets of sera from COVID-19 patients were utilized in this study (Fig. 1). The training data set was a matrix containing the relative expression of 331 proteins in 54 sera samples from 40 patients (25 non-severe and 15 severe) with 21.7% missing values. The mean age of the patients was 51.1 years and the mean body mass index (BMI) was 23.9. Severe patients exhibited a higher BMI (*p* < 0.01) and a higher incidence of hypertension and diabetes than the non-severe cases (Table 1 and Additional file 2: Table S1). In addition, two test datasets were included. The first is the TMTpro 16plex data set from our previous publication [3], containing a relative expression of 894 proteins in 21 sera samples from 21 patients (6 non-severe and 15 severe). The other is a 5-min gradient SWATH data containing the relative expression of 229 proteins in 102 sera samples from 31 German patients with 12.2% missing values [4]. The details of the protein matrix are summarized in Table 2.

**Table 1:** Information of 40 patients in the training set and all total patients

	Study total (40)	Study non-severe (25)	Study severe (15)	All total (144)	All non-severe (108)	All severe (36)
Sex (male/female)	17/23	8/17	9/6	77/67	57/51	20/16
Age	51.1 ± 17.3	48 ± 18.9	56.3 ± 13.1	47.6 ± 14.6	45 ± 14.2	55.5 ± 12.8^**##**^
BMI	23.9 ± 3.3	22.7 ± 3.2	26.1 ± 2.1**	24.2 ± 3.1	23.9 ± 3.2	25.5 ± 2.3^**##**^
Onset admission	7.5 ± 4.4	7.5 ± 4.7	7.4 ± 4.1	7 ± 4.2	6.8 ± 3.9	7.9 ± 4.9
Admission discharge	27.5 ± 8.9	27.6 ± 10	27.2 ± 7	21.6 ± 9.4	20.5 ± 9.7	24.7 ± 7.8^**#**^
Symptoms (%)						
Fever	27 (67)	12 (48)	15 (100)**	104 (72.2)	70 (64.8)	34 (94.4)^**##**^
Pharyngalgia	6 (15)	5 (20)	1 (6.7)	17 (11.8)	15(13.9)	2 (5.6)
Cough	18 (45)	12 (48)	6 (40)	65 (45.1)	47(43.5)	18 (50)
Expectoration	10 (25)	7 (28)	3 (20)	26 (18.1)	19(17.6)	7 (19.4)
Fatigue	2 (5)	1 (4)	1 (6.7)	16 (11.1)	10 (9.3)	6 (16.7)
Headache	4 (10)	2 (8)	2 (13.3)	16 (11.1)	9 (8.3)	7 (19.4)
Diarrhea	1 (2.5)	0 (0)	1 (6.7)	6 (4.2)	3 (2.8)	3 (8.3)
Chest tightness	4 (10)	2 (8)	2 (13.3)	11 (7.6)	7 (6.5)	4 (11.1)
Comorbidity (%)						
Hypertension	8 (20)	4 (16)	4 (26.7)	22 (15.3)	14 (13)	8 (22.2)
Diabetes	6 (15)	2 (8)	4 (26.7)	14 (9.7)	9 (8.3)	5 (13.9)
Hyperlipidemia	2 (5)	1 (4)	1 (6.7)	3 (2.1)	2 (1.9)	1 (2.8)
Cardiovascular disease	1 (2.5)	0 (0)	1 (6.7)	3 (2.1)	1 (0.9)	2 (5.6)
Kidney disease	1 (2.5)	0 (0)	1 (6.7)	2 (1.4)	1 (0.9)	1 (2.8)
Digestive system	3 (7.5)	2 (8)	1 (6.7)	7 (4.9)	6 (5.6)	1 (2.8)

**p < 0.01, study severe vs study non severe

^#^*p* < 0.05 and ^##^*p* < 0.01, all severe vs all non-severe

**Table 2:** Summary of the dataset used for this study

	Patients (non-severe/severe)	Samples	MS Method	Proteins	Missing (%)
Training	40 (25/15)	54	20 min SWATH	331	21.7
Test 1	21 (6/15)	21	TMTpro 16plex	894	35.5
Test 2	31 (12/19)	102	5 min SWATH	229	12.2

### Extraction of protein context features

We established a few protein context features including protein complexes, protein stoichiometric ratios in a protein complex, pathways, proteins and network degrees. A total of 868 features were obtained based on the quantification of 331 proteins (Additional file 3: Table S2). For protein complexes, two databases, namely BioPlex and CORUM, were utilized to retrieve the complex entities based on the 331 proteins. This led to identification of 27 potentially functional protein complexes from BioPlex, a database of human protein–protein interactions based on affinity purification mass spectrometry (AP-MS) [19]. In addition, 16 protein complexes were identified from the CORUM database, a manually curated and experimentally characterized protein complexes repository [18]. Therefore, BioPlex and CORUM together led to 43 protein complexes (Additional file 4: Table S3). For each protein complex, we computed the ratio of each protein pair, leading to 105 protein ratios (Additional file 4: Table S3). Subsequently, 58 pathway features were enriched by 71 differentially expressed proteins (Limma, adjust *p* < 0.05) between severe and non-severe patients by G:profiler [21]. Thus, we compiled a feature list containing 43 complexes, 71 differentially expressed proteins and 58 enriched pathways (Additional file 4: Table S3), which were utilized as input features for machine learning to stratify COVID-19 patients. Additionally, protein network degrees, which reveal the co-expression relationships with other proteins [16], were also applied as one type of feature. A total of 331 protein degrees were obtained as features (Additional file 3: Table S2). Finally, we focused on differential features between severe and non-severe patients using limma (p value < 0.05). A total of 192 differential features were obtained, including 7 complexes, 16 protein stoichiometry ratios, 27 pathways, 71 proteins, and 71 protein network degrees (Additional file 5: Table S4).

### Classification of severe patients using machine learning

**Fig. 2:**
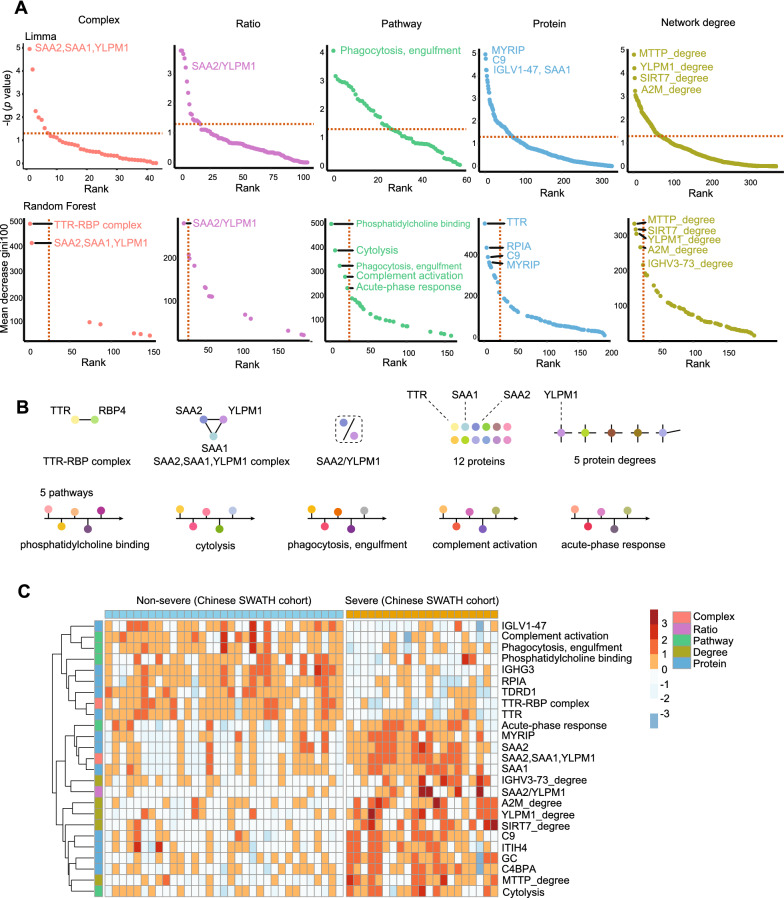
The selected features for classifying COVID-19. **A** All Identified features ranked by log *p* value; **B** The top 25 features identified; **C** The heatmap of top 25 features in the training set

As shown in Fig. 2A, all the 192 identified features by Limma were ranked by log-scaled *p* values. To further identify biomarkers for the classification of severe cases, a random forest machine learning model based on the above-mentioned features was applied to the training cohort. The fit of the model was evaluated by the area under the curve (AUC) (Fig. 2A). The best classifier contained the top twenty-five features, including two complexes, one stoichiometric ratio, five pathways, twelve proteins, and five network degrees (Fig. 2B, Additional file 1: Fig. S1). The changes of the top 25 features in sera of severe cases (Chinese SWATH cohort) are visualized in a heatmap (Fig. 2C).

**Fig. 3:**
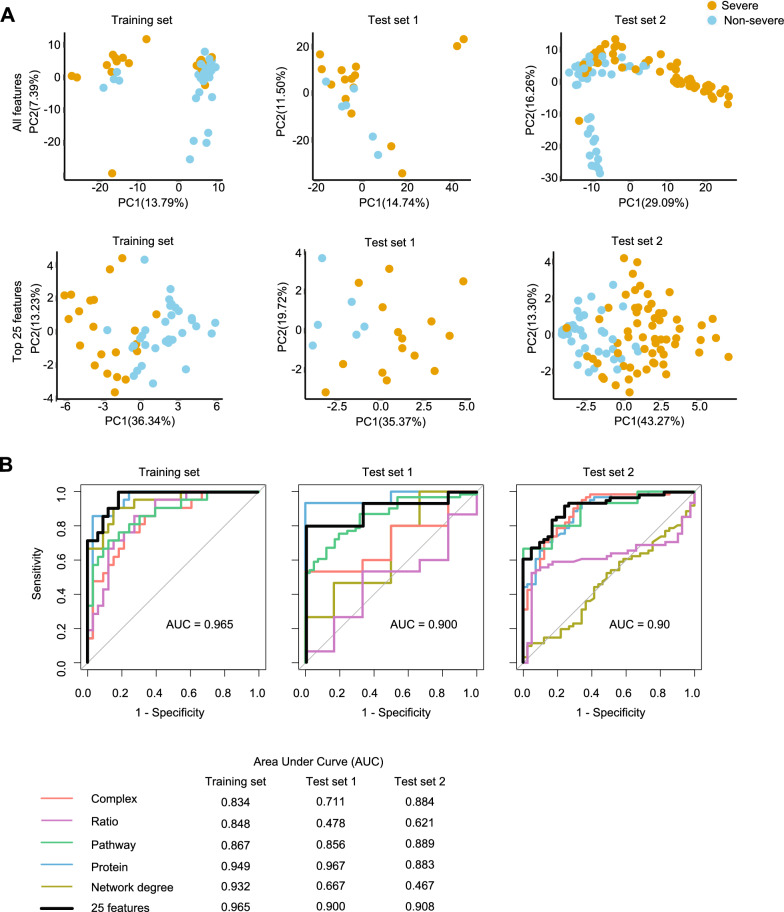
The performance of the machine learning model. **A** The PCA map of the training set and test sets using all features; **B** The comparison of AUC between the model with five types of features and the model with only one type of feature in the training set, test set 1, and the test set 2

As shown in Fig. 3A, severe and non-severe cases in training set and two independent test sets were not well separated when all features were used. However, by using the top 25 features, the severe and non-severe cases in the training set and two test sets can be well distinguished. We next tested this model in an independent TMT-based proteomic data set containing 6 non-severe and 15 severe cases (Test set 1). The model achieved an AUC of 0.900 in this dataset (Fig. 3B). It should be noted that not all the top 25 features were used in test sets due to some missing features. Actually, 18 features, including two complexes, five pathways, nine proteins, and two network degrees, were identified in the test data set 1. Next, this classifier was further evaluated using another independent SWATH data set from 12 non-severe and 19 severe German patients (Test set 2) [4]. In total, 16 features, including two complexes, five pathways, eight proteins, and one network degree were identified in the test data set 2, leading to an AUC of 0.908 (Fig. 3B). The AUC results reveal that, besides proteins, protein complexes, stoichiometric ratios, pathways and protein degrees could be potential biomarkers for stratification of COVID-19 patients.

To validate whether the selected features were optimal for our classifier, we built 200 models with random features and validated them with the TMT data set and the SWATH-based German cohort dataset. The median AUC of these models is 0.756, which is significantly lower than the AUC of the model with top 25 features (0.900), indicating the superiority of our selected features.

### Modeling with only one type of feature

To explore whether the machine learning models with five different types of features are superior to the models with only one type of feature, we trained models with only one type of feature and tested them with the TMT-based proteomics data set (test set 1) and the SWATH-based German cohort data set (test set 2). As shown in Fig. 3B, for the training set and test set 2, the AUC values for the model with all five types of features were 0.965 and 0.908, respectively, which were better than the AUC of the model only with proteins as the feature (0.949 and 0.883, respectively). For test set 1, the model with all types of features reached an AUC of 0.900, which was slightly lower than that of the model only with proteins. This may be because that test set 1 is based on the TMT-tagging data acquisition mode, which is different from the SWATH data of the training data set and test set 2. In addition, the difference in sample size may have also contributed.

The AUC for the model with only the protein network degree as features reached 0.932, 0.667, and 0.467 in the training set and the two test sets, respectively, indicating that it performs well in the training set, but not in the two test sets (Fig. 3B). Similar observation was found in the model with only the protein complex ratio as a feature (Fig. 3B). In addition, in the training set and two test sets, the AUC values of the model with five types of features were all better than those of the models with only complex or pathway. These findings together consolidate the benefit of integrating multiple model features for COVID-19 patient stratification.

## Discussion

The highlight of this study is that we integrated five types of features including protein complexes, protein stoichiometric ratios, pathways, network degrees, and proteins, rather than using purely individual proteins, to build machine learning models for disease classification. Twenty-five predictive markers were identified to stratify COVID-19. Our work demonstrates that integrating protein expression levels with protein context improves COVID-19 patient stratification.

**Fig. 4:**
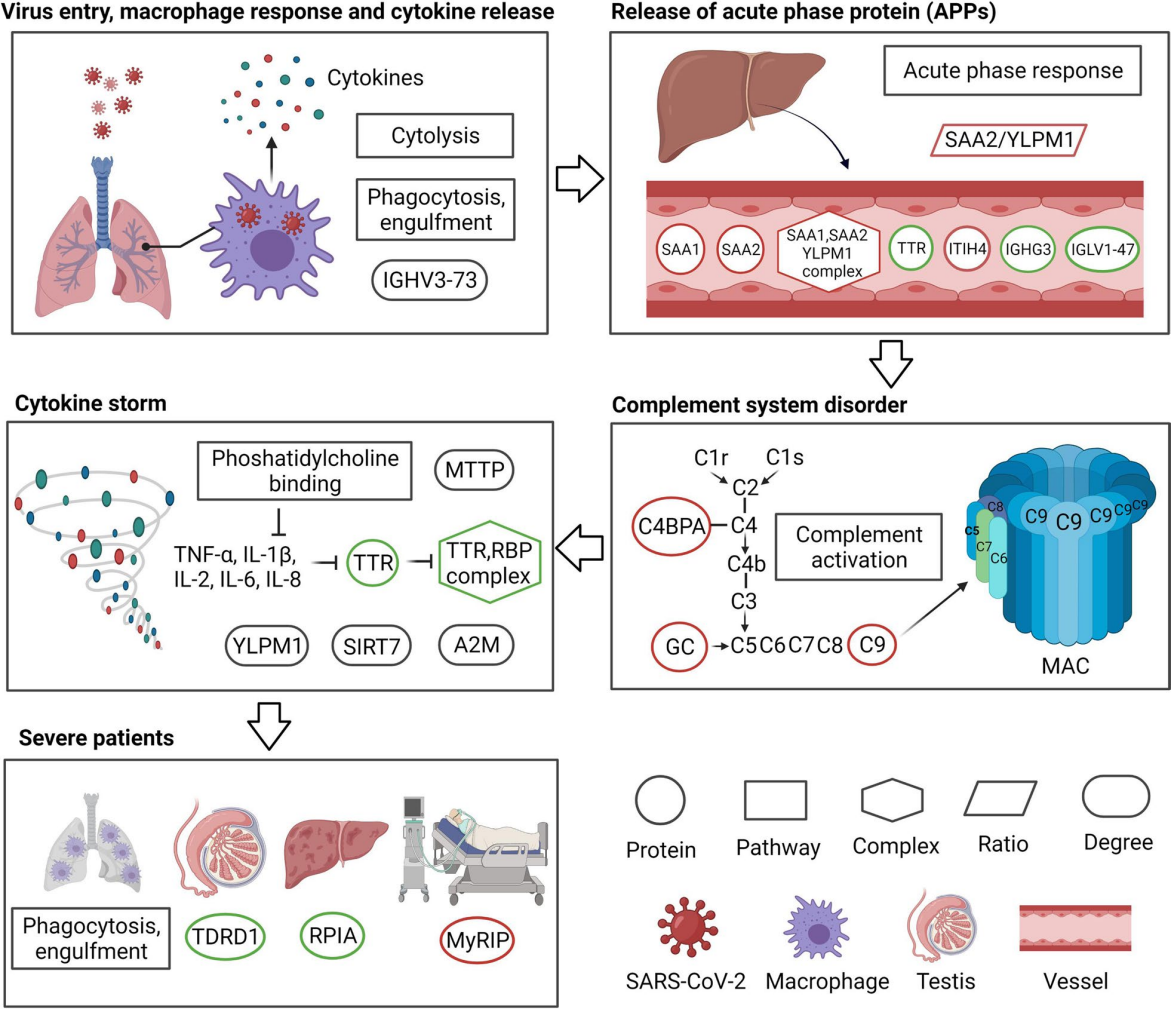
The biological interpretation of the top 25 features. MAC, membrane attack complex. Red border, upregulation; green boarder, downregulation

The 25 features highlighted by our analysis are all associated with the pathogenesis of COVID-19. As shown in Fig. 4, after the SARS-CoV-2 enters the alveolar, the macrophages subsequently phagocytose the virus and release cytokines, resulting in the release of acute phase proteins (APPs) from the liver [22]. These APPs stimulate the complement system response [23]. However, in severe cases, the complement system reacts abnormally, which can potentially trigger a cytokine storm [24, 25]. On one hand, cytokine storm leads to multi-organ damages, such as damages to the liver and testis [26]. On the other hand, more macrophages are recruited from the peripheral blood to the lungs, causing alveolar macrophage infiltration, lung damage, and respiratory failure [27].

Several studies have reported predictive blood markers for severe cases, such as ITIH4 [28, 29], M-CSF, CCL3 and CCL4 [30], as well as CMAs [31]. Studies utilizing MS-based proteomics also have found that proteins associated with complement system, acute phase protein response, inflammation system, macrophage dysregulation, antibody response, and coagulation system are altered in severe COVID-19 cases [3, 4, 7, 9, 32], which have also been confirmed by other proteomic approaches [33–35]. In this study, we found a complex, two pathways, seven proteins and one network degree are involved in the complement system, acute phase proteins and inflammation, including “SAA1, SAA2, YLPM1”, “complement activation”, “acute-phase response”, IGHG3, SAA1, SAA2, IGLV1-47, C9, ITIH4, C4BPA and IGHV3-73 (Additional file 1: Fig. S1). In addition, one pathway (phagocytosis, engulfment) associated with macrophage dysregulation was identified as a key feature. Our data uncovered previously hidden COVID-19-associated proteome context information.

Our study also identified other molecular features in severe patients. Several transport proteins were upregulated. Vitamin D-binding protein (GC) enhances the activity of C5a in the complement system [36], which may induce cytokine storms. MyRIP, another transport protein, participates in melanosomes and produces pigmented melanin to skins [37]. The upregulation of MyRIP may be related to skin hyperpigmentation in severe patients [38]. Transthyretin (TTR) is a marker for inflammation and a negative acute-phase reactant. Reduced TTR has been reported to be associated with acute-phase response induced by inflammation, and TTR is also a malnutrition marker, suggesting nutritional disorders in severe cases [39]. TTR-RBP complex consists of TTR and retinol-binding protein 4 (RBP4), and the upregulation of TTR-RBP complex suggests an improved inflammation state [40]. In this study, both TTR and TTR-RBP complexes decreased in the sera of severe cases, suggesting a more intense acute response and inflammatory state.

Notably, some proteins associated with the complement system were also altered in severe cases. Abnormal response of complement system can trigger cytokine storm, which can further develop into severe cases [24, 41]. Carvelli et al. found that C5 was the main effector of abnormal complement system, and blockade of C5 could prevent excessive lung inflammation [42]. Complement protein C3 was also associated with fatal outcome of COVID-19 [25]. Different from previous studies, we found that C9, another protein in the complement system, was elevated in severe cases, suggesting that it may also be a marker or potential therapeutic target. In addition, GC, which activates the activity of C5 [36], was also upregulated. C4BPA associated with C4 activity was abnormally expressed [43] (Fig. 4). In addition to changes in proteins associated with complement system, RPIA was downregulated in severe cases, which may indicate an impaired glucose metabolism and liver damage. The Tudor domain-containing protein 1 (TDRD1), which plays a central role in spermatogenesis [44], was also downregulated, which may contribute to impaired testis functions observed in severe cases [26].

In addition to proteins, other types of protein context feature further shed light on the mechanism of severe COVID-19 cases. Cytolysis pathway is induced after viral infection and serves as a clearance mechanism for infected cells [45]. The alteration of the phosphatidylcholine binding pathway may contribute to the inflammatory process [46]. The increased ratio of SAA2/YLPM1 in the "SAA2, SAA1, YLPM1" complex in severe cases may be due to upregulation of SAA2 (sera amyloid A-2 protein) and downregulation of YLPM1 (YLP motif-containing protein 1, Additional file 1: Fig. S1), revealing an acute-phase response and an enhanced repair of inflammation-induced telomere shortening [47]. The network degree changes of some proteins were associated with cytokine storm. Immunoglobulin heavy variable 3-73 (IGHV3-73) participates in antigen recognition [48]. MTTP stimulates phosphatidylcholine transport [49]. Alpha-2-macroglobulin (A2M) influences cytokines signaling [50], and SIRT7 suppresses inflammation [51]. Since network degree suggests the co-expression associations with other proteins, the network degree changes of these proteins also uncorvered systematic molecular changes in severe cases. Our study showed that the predictive result of the model with five different features was better than that of the model with one single feature (Fig. 3B), suggesting the benefits of integrating multiple protein context in disease prediction and stratification.

Some limitations of this study should be noted. There were missing features in the two test sets. Seven features were not included in the TMT data, and nine features were excluded in the German cohort data. Median value of all the valued features were used to impute these missing features. The sample size of the training set is limited. Nevertheless, the model achieved satisfactory AUCs in these independent tests. Neither these limitations compromise the major conclusion of this study that integrating protein context information improves COVID-19 severity classification. Moreover, the protein complex information was obtained from cellular complexes, meaning that not all the complexes are necessarily formed in the serum, which needs to be verified by future research. Finally, building ratios may create an overfitting danger, but this can be avoided by building models with other types of features together.

## Conclusion

Protein complexes, stoichiometric ratios, pathways and network degrees could be used as biomarkers to identify severe cases. Our present study confirms some of the previously reported molecular changes and identifies some new features that may contribute to understand the pathogenesis of COVID-19.

## Supplementary Information

**Additional file 1: Fig. S1.** Expression of the 25 features in COVID-19 sera. (A) log2-scaled protein intensity; (B) Protein complex features indicated by z score and stoichiometric ratio of SAA2/YLPM1; (C) z scores of pathways; (D) z scores of network degree features.

**Additional file 2: Table S1.** Information of 40 patients in the training set and all total patients.

**Additional file 3: Table S2.** The total 868 features based on 331 proteins.

**Additional file 4: Table S3.** The complexs, differential proteins and enriched pathways used as features.

**Additional file 5: Table S4.** The total 192 differential features between severe and non-severe patients.

## Data Availability

All data generated or analyzed during this study are included in this published article.

## Abbreviations

APPs: Acute phase proteins
GC: Vitamin D-binding protein
TTR: Transthyretin
RBP4: Retinol-binding protein 4
TDRD1: Tudor domain-containing protein 1
SAA2: Sera amyloid A-2 protein
A2M: Alpha-2-2 macroglobulin
IGHV3-73: Immunoglobulin heavy variable 3-73
