# Supplementary figures and images for: Integration of protein context improves protein-based COVID-19 patient stratification
Source: Clin Proteomics. 2022 Aug 11;19:31. doi: 10.1186/s12014-022-09370-0 (PMC9366758; doi:10.1186/s12014-022-09370-0)

Figure S1

A

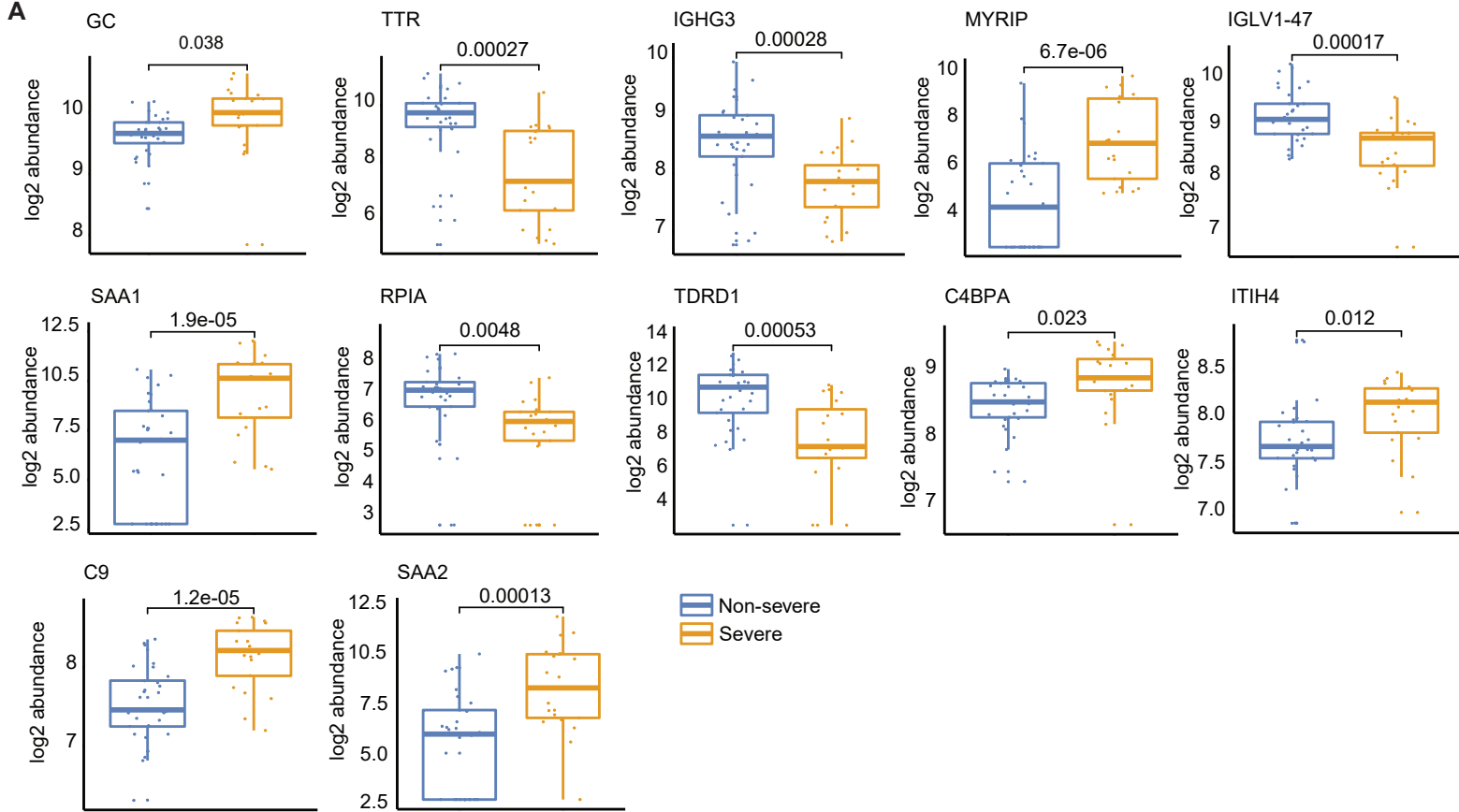

B

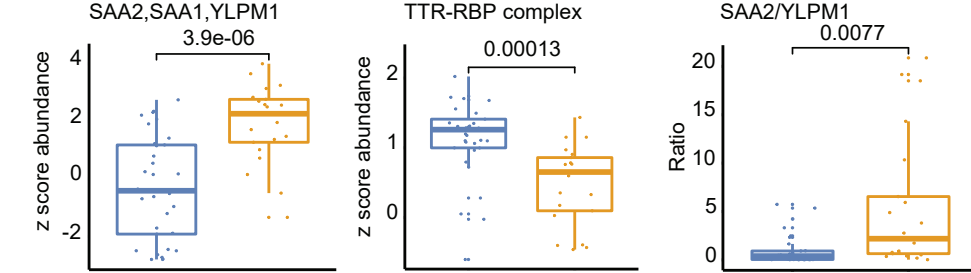

C

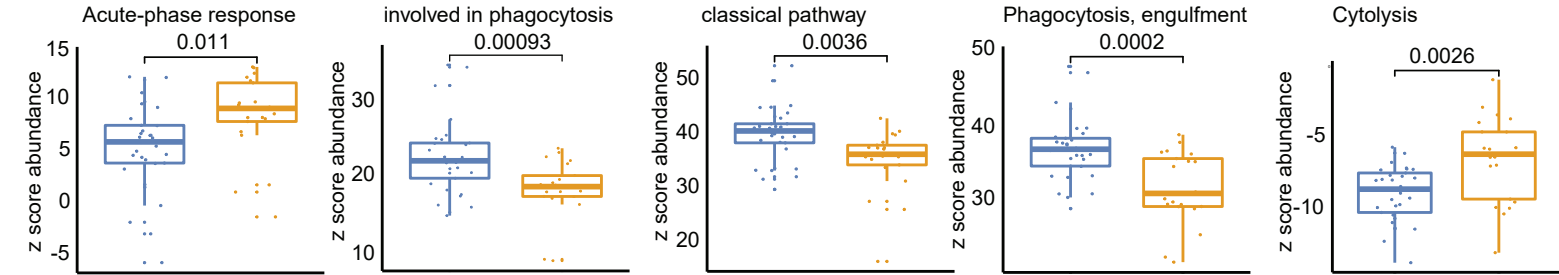

D

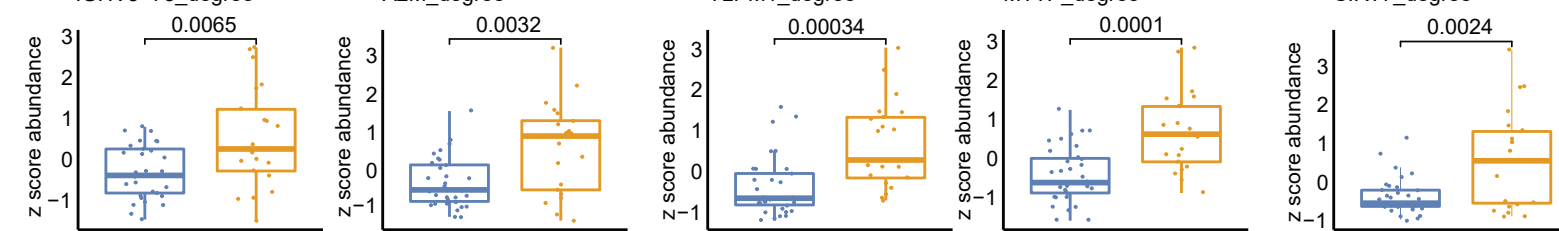

Supplement: Supplementary file 1 — Additional file 1: Fig. S1. Expression of the 25 features in COVID-19 sera. (A) log2-scaled protein intensity; (B) Protein complex features indicated by z score and stoichiometric ratio of SAA2/YLPM1; (C) z scores of pathways; (D) z scores of network degree features. [file 12014_2022_9370_MOESM1_ESM.pdf]
