# Supplementary material for: Integration of protein context improves protein-based COVID-19 patient stratification
Source: Clin Proteomics. 2022 Aug 11;19:31. doi: 10.1186/s12014-022-09370-0 (PMC9366758; doi:10.1186/s12014-022-09370-0)
Supplement: Supplementary file 2 — Additional file 2: Table S1. Information of 40 patients in the training set and all total patients. [file 12014_2022_9370_MOESM2_ESM.docx]

**Supplementary Table S1. Information of 40 patients in the training set and all total patients.**

|  | **Study**  **total**  **(40)** | **Study**  **Non severe**  **(25)** | **Study**  **severe**  **(15)** | **All**  **total**  **(144)** | **All**  **non severe (108)** | **All**  **severe**  **(36)** |
| --- | --- | --- | --- | --- | --- | --- |
| Age  Median (IQR) | 52.5  (40.8-63.2) | 50  (37-56) | 55  (49-66.5) | 47  (38-56) | 44.5  (37-54) | 55  (49.2-65) |
| Age  (Range) | 4-86 | 4-86 | 33-77 | 4-86 | 4-86 | 30-79 |
| BMI  Median (IQR) | 23.9  (21.4-26.7) | 22  (20.8-24.4) | 26.6  (24.7-27.3) | 24.4  (22-26.4) | 23.8  (21.5-26) | 25.5  (24.3-27.1) |
| BMI (Range) | 16-29.6 | 16-29.6 | 22.7-28.7 | 16-31.3 | 16-31.2 | 21.6-31.3 |
| Onset admission  Median (IQR) | 7.5  (3.8-10.2) | 6  (4-10) | 8  (3.5-10.5) | 6  (4-10) | 6  (4-9) | 7.5  (4-11) |
| Onset admission  (Range) | 1-17 | 1-17 | 2-14 | 1-24 | 1-18 | 1-24 |
| Admission discharge  Median (IQR) | 27  (21.8-34.5) | 29  (21-38) | 26  (22.5-33) | 21.5  (13-28) | 20  (13-27) | 23  (19.8-31.2) |
| Admission discharge  (Range) | 6-41 | 6-41 | 17-40 | 6-44 | 6-44 | 9-40 |
| Comorbidity (%) | | | | | | |
| Cerebrovascular diseases | 0 (0) | 0 (0) | 0 (0) | 3 (2.1) | 2 (1.9) | 1 (2.8) |
| Chronic bronchitis | 1 (2.5) | 1 (4) | 0 (0) | 4 (2.8) | 4 (3.7) | 0 (0) **^#^** |
| Tuberculosis | 0 (0) | 0 (0) | 0 (0) | 4 (2.8) | 4 (3.7) | 0 (0) **^#^** |
| Malignant tumor | 0 (0) | 0 (0) | 0 (0) | 2 (1.4) | 1 (0.9) | 1 (2.8) |
| Thyroid disease | 2 (5) | 0 (0) | 2 (13.3) | 5 (3.5) | 2 (1.9) | 3 (8.3) |
| Syphilis | 1 (2.5) | 0 (0) | 1 (6.7) | 2 (1.4) | 1 (0.9) | 1 (2.8) |
| Hepatitis | 2 (5) | 2 (8) | 0 (0) | 7 (4.9) | 5 (4.6) | 2 (5.6) |
| Gout | 1 (2.5) | 0 (0) | 1 (6.7) | 1 (0.7) | 0 (0) | 1 (2.8) |
| Basic diseases | 20 (50) | 10 (40) | 10 (66.7) | 58 (40.3) | 40 (37) | 18 (50) |
| Cardiovascular basic diseases | 13 (32.5) | 6 (24) | 7 (46.7) | 34 (23.6) | 22 (20.4) | 12 (33.3) |
| Thyroid disease | 1 (2.5) | 0 (0) | 1 (6.7) | 4 (2.8) | 2 (1.9) | 2 (5.6) |
| Chest CT (%) | 40 (100) | 25 (100) | 15 (100) | 143 (99.3) | 107 (99.1) | 36 (100) |
| Other indexes | | | | | | |
| Heart rate  (Mean ± SD) | 83.6±12.3 | 82 ±11.8 | 85.8±13.2 | 83.5±12.9 | 83.2±12.9 | 84.1±12.7 |
| Heart rate  Median (IQR) | 80.5  (75-91.8) | 78  (75-89) | 86  (76-97) | 82  (75-91) | 82  (75-90) | 83.5  (75-93.5) |
| Heart rate  (Range) | 57-115 | 64-115 | 57-105 | 57-147 | 57-147 | 57-106 |
| Breath rate  Mean ± SD | 18.6±1.4 | 18.9±0.9 | 18.2±2 | 18.9±1.4 | 18.9±1.3 | 18.8±1.6 |
| Breath rate  Median (IQR) | 19  (18-19) | 19  (18-19.5) | 18  (18-19) | 19  (18-20) | 19  (18-20) | 19  (18-20) |
| Breath rate  (Range) | 12-21 | 18-21 | 12-20 | 12-26 | 16-26 | 12-22 |
| Arterial partial pressure  (Mean ± SD) | 98±33.2 | 102.6±25 | 90.8±43.3 | 97.6±29.9 | 102.1±28.1 | 81.7±31.3**^##^** |
| Arterial partial pressure  Median (IQR) | 91  (78.2-109.2) | 95  (86-116) | 76  (63.5-87) | 93  (80-104) | 94.1  (84-108) | 73  (64-81) |
| Arterial partial pressure (Range) | 62-195 | 65-156 | 62-195 | 51-222 | 65-222 | 51-195 |
| Amyloid A  (Mean ± SD) | 353±488 | 154±380 | 657± 488**^**^** | 214±331 | 121.8±225 | 477.7±432.7**^##^** |
| Amyloid A  Median (IQR) | 123.5  (20.9-490.1) | 31.5  (8.8-123.5) | 550.8  (269-1035.3) | 101.8  (18.2-193) | 44.6  (10.2-137.9) | 400.3  (114.8-703.2) |
| Amyloid A  (Range) | 5.8-1831 | 5.8-1831 | 21.1-1580.6 | 5-1831 | 5-1831 | 5-1580.6 |
| Retinol binding protein  (Mean ± SD) | 31.1±10.7 | 31.9±7.4 | 29.7±14.8 | 32.1±9.5 | 33.3±8.6 | 28.6±11.2**^#^** |
| Retinol binding protein  Median (IQR) | 30  (24.6-35.3) | 31.5  (28.8-37) | 24.6  (23.5-30.2) | 31.3  (25.5-36.9) | 32.5  (29-38) | 26.1  (23.9-32) |
| Retinol binding protein  (Range) | 14.7-76.3 | 18.3-50.4 | 14.7-76.3 | 8.3-76.3 | 14.5-64.8 | 8.3-76.3 |
| Arterial blood sugar  (Mean ± SD) | 9.2 ± 5.2 | 7.7 ± 3 | 11.7 ± 6.9**^*^** | 8.2 ± 3.8 | 7.7 ± 3.1 | 9.6 ± 5.1**^#^** |
| Arterial blood sugar  Median (IQR) | 7  (6-10.6) | 6.8  (5.8-8.2) | 9.1  (6.3-16.1) | 7.1  (5.8-9.1) | 6.8  (5.6-8.6) | 8.1  (6-10.6) |
| Arterial blood sugar  (Range) | 4.7-26 | 4.7-15.8 | 4.7-26 | 2.8-26 | 2.8-19.2 | 4.7-26 |
| Hemoglobin  (Mean ± SD) | 14.4±2.5 | 14.2±2.5 | 14.7±2.5 | 14.8±2.7 | 14.8±2.7 | 14.8±2.9 |
| Hemoglobin  Median (IQR) | 14.2  (12.9-15.8) | 14.1  (12.4-15.8) | 14.5  (13.3-15.8) | 14.5  (12.9-16.8) | 14.5  (12.9-16.4) | 14.5  (12.9-17.2) |
| Hemoglobin  (Range) | 8.3-20.1 | 9.9-20.1 | 8.3-19.5 | 8.3-21.1 | 9.2-21.1 | 8.3-20.5 |
| Hematocrit  (Mean ± SD) | 44.2±8.4 | 43±7.7 | 46±9.3 | 46±9.2 | 46±9 | 46.1±9.9 |
| Hematocrit  Median (IQR) | 43  (39-48.5) | 42.5  (37.8-48) | 46  (41.5-50) | 45  (39-52.5) | 45  (39.5-52.5) | 46  (39-52.2) |
| Hematocrit  (Range) | 25-65 | 30-61 | 25-65 | 25-65 | 28-65 | 25-65 |
| CK  (Mean ± SD) | 100.8±109 | 58.8±25.6 | 168±152.1**^*^** | 100.3±108 | 77.5±63.1 | 167.2±169.8**^##^** |
| CK  Median (IQR) | 69  (42.5-91) | 59.5  (37.5-76.2) | 97  (66-193.5) | 68.5  (44.2-88.8) | 62.5  (43.2-80) | 93.5  (66.5-185.8) |
| CK  (Range) | 23-481 | 23-119 | 28-481 | 18-688 | 23-355 | 18-688 |
| CKMB  (Mean ± SD) | 1.4±1.4 | 1.1±1.4 | 1.9±1.3 | 1.1±1.1 | 1±1 | 1.5±1.1**^#^** |
| CKMB  Median (IQR) | 0.7  (0.5-2.1) | 0.7  (0.5-0.8) | 2  (0.6-2.6) | 0.7  (0.4-1.3) | 0.6  (0.4-1.1) | 1.3  (0.6-2.4) |
| CKMB  (Range) | 0.2-6 | 0.3-6 | 0.2-4.6 | 0.2-6.2 | 0.2-6.2 | 0.2-4.6 |
| LDH  (Mean ± SD) | 231.9±91.6 | 188.8±42.9 | 300.9±107**^**^** | 220.5±82.2 | 193.2±39.6 | 301.9±116.5**^##^** |
| LDH  Median (IQR) | 207  (168.5-258) | 173.5  (154-215.8) | 270  (231-343.5) | 200  (170.5-239.5) | 187  (163-216.2) | 274  (226-324) |
| LDH  (Range) | 137-496 | 137-310 | 167-496 | 120-721.6 | 120-338 | 167-721.6 |
| MYO  (Mean ± SD) | 0.01±0.02 | 0.01±0.01 | 0.02±0.03 | 0.01±0.01 | 0.01±0.01 | 0.02±0.02**^#^** |
| MYO  Median (IQR) | 0.01  (0.01-0.01) | 0.01  (0-0.01) | 0.01 (0.01-0.03) | 0.01  (0-0.01) | 0.01  (0-0.01) | 0.01  (0.01-0.01) |
| MYO  (Range) | 0-0.09 | 0-0.04 | 0-0.09 | 0-0.09 | 0-0.04 | 0-0.09 |
| TNT 1  (Mean ± SD) | 56.8±73.5 | 33±59 | 90±80.6**^*^** | 36.8±46.1 | 26.8±31.4 | 65.6±66.3**^##^** |
| TNT 1  Median (IQR) | 22.4  (15.4-53.1) | 15.9  (13.8-22.1) | 44.9  (26.1-134.9) | 22.4  (14.4-35.5) | 18.4  (13.6-28.3) | 34.4  (24.4-76.8) |
| TNT 1  (Range) | 8.4-281.9 | 8.4-281.9 | 16.3-227.6 | 7.5-281.9 | 7.5-281.9 | 11.6-227.6 |

^*^ *P* < 0.05, ^**^ *P* < 0.01, study severe vs study non severe; ^#^ *P* < 0.05, ^##^ *P* < 0.01, all severe vs all non-severe.
